# SYSTEMS BIOLOGY OF HUMAN AGING - NETWORK MODEL 2019

**DOI:** 10.1093/geroni/igz038.3527

**Published:** 2019-11-08

**Authors:** John D Furber

**Affiliations:** Legendary Pharmaceuticals, Gainesville, Florida, United States

## Abstract

This network schema is presented to aid in conceptualizing the many processes of aging, the causal chains of events, and the interactions among them, including feedback and vicious cycles. Contemplation of this network suggests promising intervention points for therapy development. This diagram is maintained on the Web as a reference for researchers and students. Content is updated as new information comes to light. www.LegendaryPharma.com/chartbg.html At first glance the network looks like a complicated web. However, as a conceptual summary, in one view, we can see how the many biogerontological processes relate to each other. Importantly, examination of these relationships allows us to pick out reasonably plausible causal chains of events. Within these chains, we can see age-related changes or accumulations that appear to be promising targets for future therapy development. Especially harmful is damage to the body's regeneration and repair systems, because they normally repair damage to other structures and systems. The many observable signs of human senescence have been hypothesized by various researchers to result from several primary causes. Inspection of the biochemical and physiological pathways associated with age-related changes and with the hypothesized causes reveals several parallel cascades of events that involve several important interactions and feedback loops. This network model includes both intracellular and extracellular processes. It ranges in scale from the molecular to the whole-body level. Effects due to externalities, lifestyle, environment, and proposed interventions are highlighted around the margins of the network.

